# Supplementary material for: Therapeutic Effects of Quetiapine and 5-HT1A Receptor Agonism on Hyperactivity in Dopamine-Deficient Mice
Source: Int J Mol Sci. 2022 Jul 4;23(13):7436. doi: 10.3390/ijms23137436 (PMC9266854; doi:10.3390/ijms23137436)
Supplement: Supplementary file 1 [file ijms-23-07436-s001.zip › ijms-1787110 Supplementary Figures.pdf]

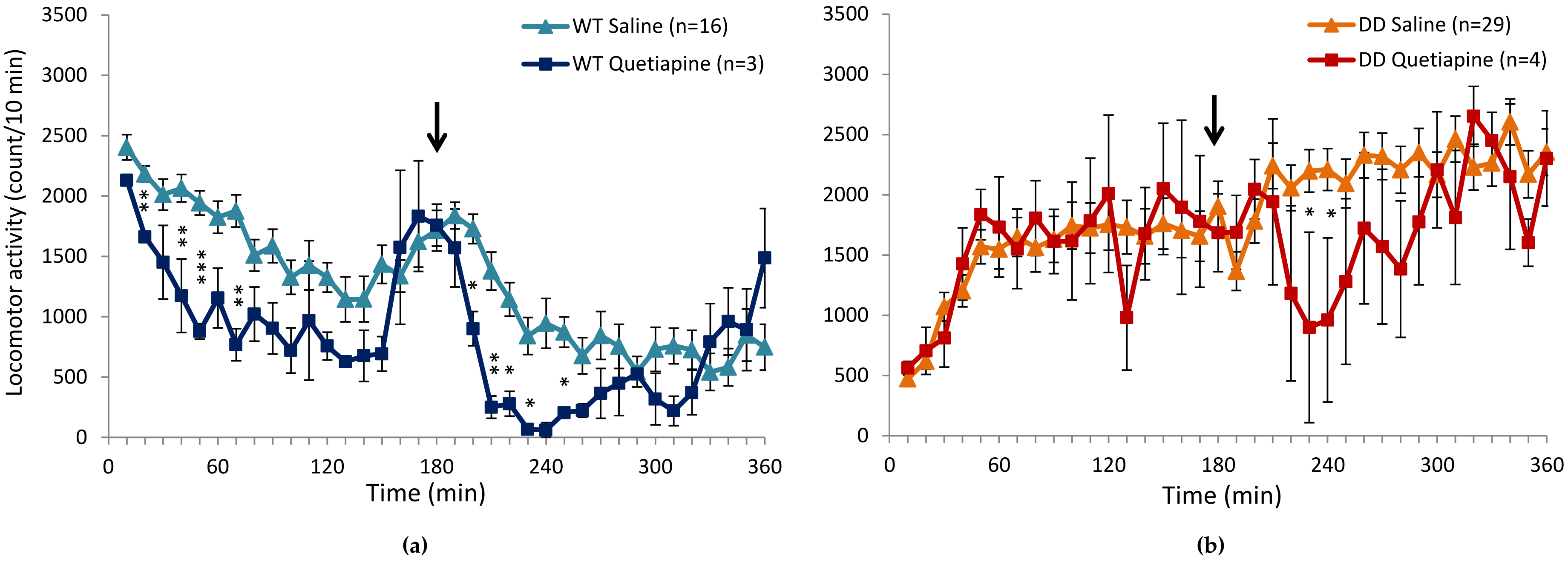

Figure S1. Changes in locomotor activity induced by quetiapine 10 mg/kg. (a, b) Changes in locomotor activity in (a) WT and (b) DD mice following quetiapine and saline administration. Refer to graph for number. The arrows indicate the drug injection time. *p < 0.05, **p < 0.01, ***p < 0.001, compared with saline (Student's t-test). The data are expressed as mean ± SEM. WT, wildtype mice; DD, dopamine-deficient mice.

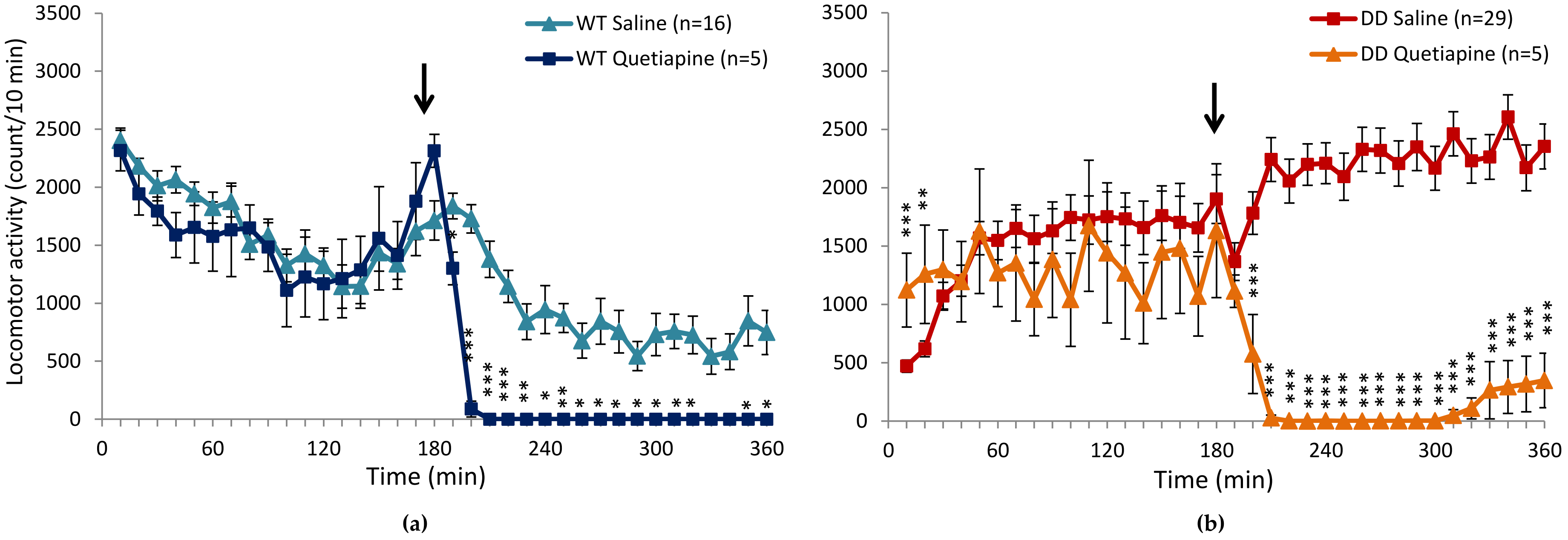

Figure S2. Changes in locomotor activity induced by quetiapine 40 mg/kg. (a, b) Changes in locomotor activity in (a) WT and (b) DD mice following quetiapine and saline administration. Refer to graph for number. The arrows indicate the drug injection time. *$p < 0.05$, **$p < 0.01$, ***$p < 0.001$, compared with saline (Student's t-test). The data are expressed as mean ± SEM. WT, wildtype mice; DD, dopamine-deficient mice.

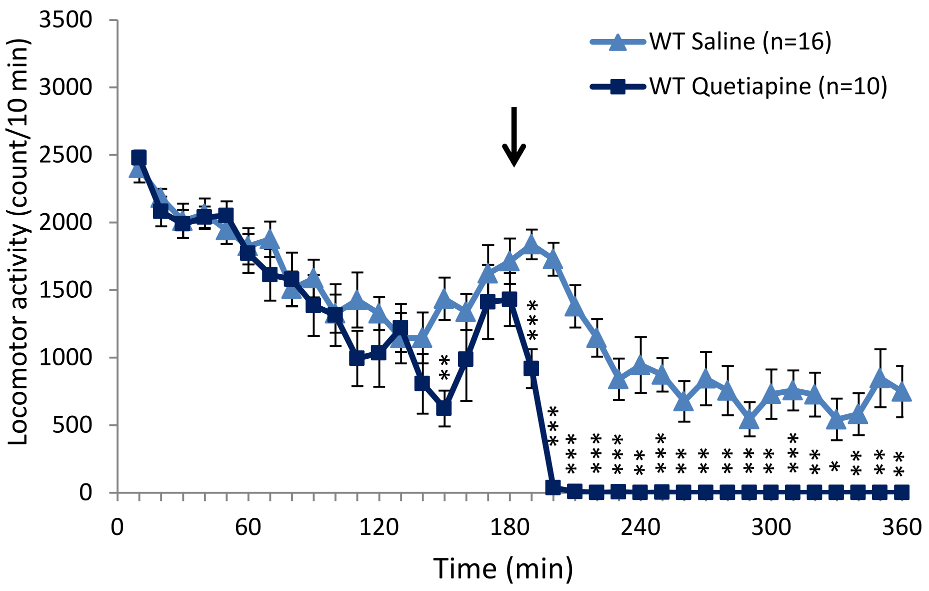

(a)

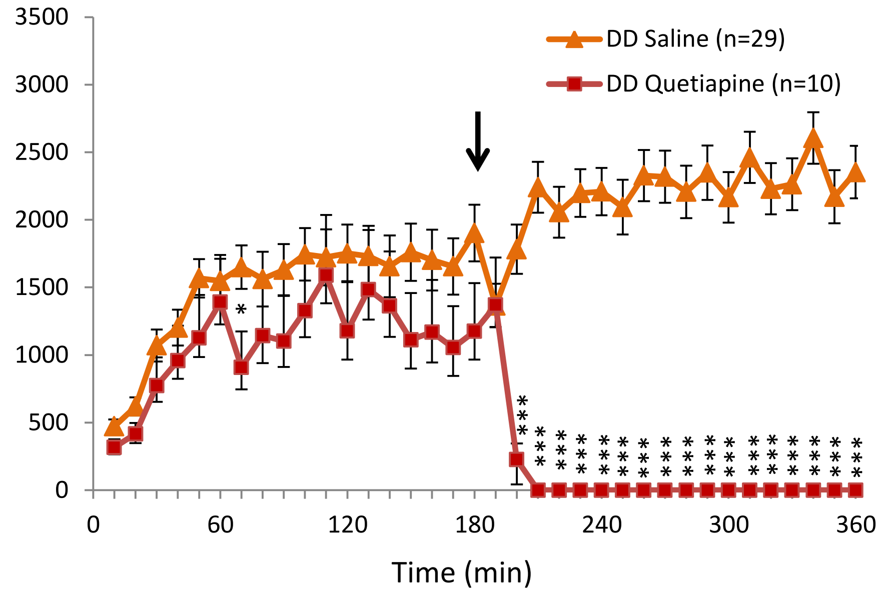

(b)

Figure S3. Changes in locomotor activity induced by quetiapine 80 mg/kg. (a, b) Changes in locomotor activity in (a) WT and (b) DD mice following quetiapine and saline administration. Refer to graph for number. The arrows indicate the drug injection time. *$p < 0.05$, **$p < 0.01$, ***$p < 0.001$, compared with saline (Student's t-test). The data are expressed as mean ± SEM. WT, wildtype mice; DD, dopamine-deficient mice.

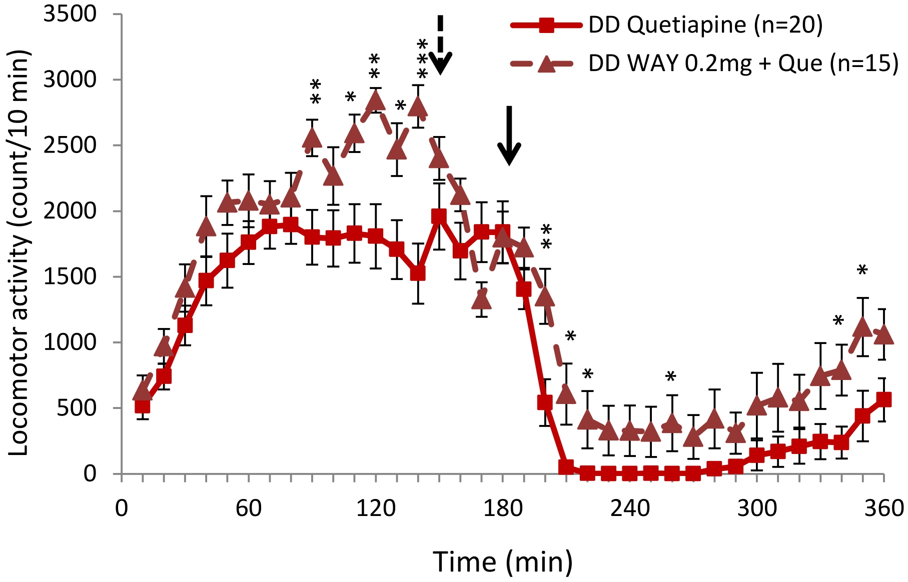

Figure S4. Changes in locomotor activity induced by WAY100635 0.2 mg/kg in DD mice 30 min before quetiapine administration. Refer to graph for number. The solid arrows indicate the quetiapine injection time. The dashed arrows indicate the WAY100635 administration time. *p < 0.05, **p < 0.01, ***p < 0.001, compared with quetiapine (Student's t-test). The data are expressed as mean ± SEM. WT, wildtype mice; DD, dopamine-deficient mice.

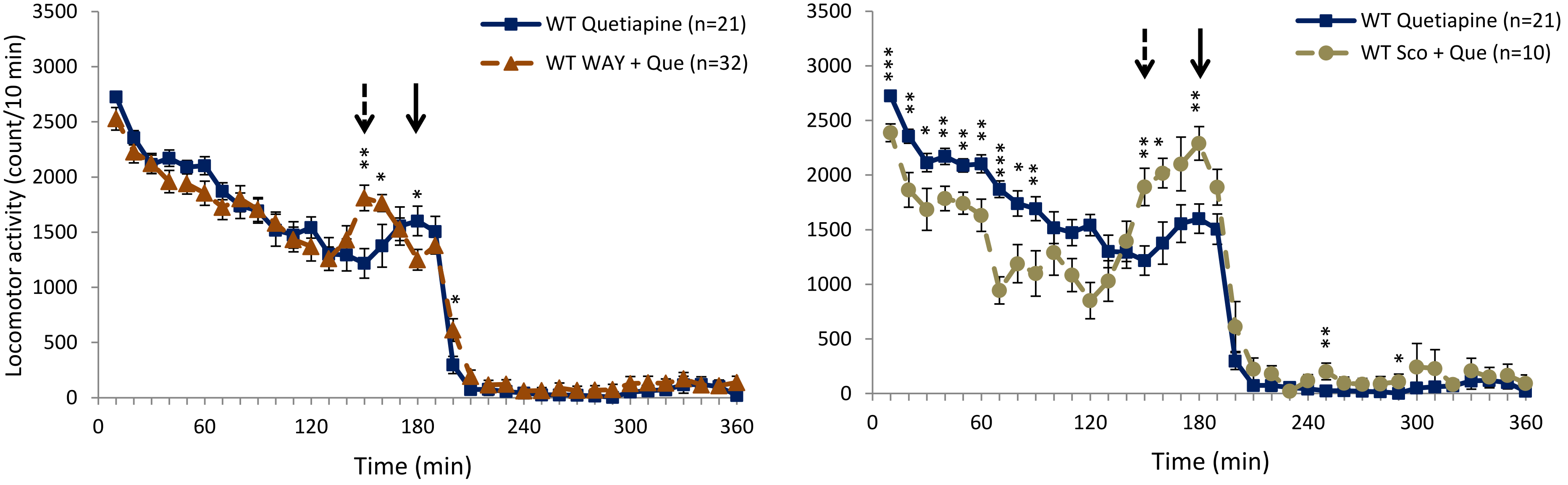

(a) (b)

Figure S5. Effects of quetiapine on locomotor activity when 5-HT1A and cholinergic receptor activity is suppressed. (a, b) Changes in locomotor activity induced by (a) WAY100635 1 mg/kg and (b) scopolamine 0.1 mg/kg in WT mice 30 min before quetiapine administration. Refer to graph for number. The solid arrows indicate the quetiapine injection time. The dashed arrows indicate the WAY100635 and scopolamine administration time. *$p < 0.05$, **$p < 0.01$, ***$p < 0.001$, compared with quetiapine (Student's t-test). The data are expressed as mean ± SEM. 5-HT, 5-hydroxytryptamine (serotonin); WT, wildtype mice.
